# Maintenance therapy for recurrent epithelial ovarian cancer: current therapies and future perspectives – a review

**DOI:** 10.1186/s13048-019-0579-0

**Published:** 2019-11-04

**Authors:** Sudeep Gupta, Shona Nag, Shyam Aggarwal, Amit Rauthan, Narayanankutty Warrier

**Affiliations:** 1 Advanced Centre for Treatment, Research & Education in Cancer, Tata Memorial Centre, Room 305, 3rd Floor, Paymaster Shodhika, Navi Mumbai, Mumbai, 410210 India; 2 Jehangir Hospital, Pune, India; 3 Sir Gangaram Hospital, New Delhi, India; 4 Manipal Hospital, Bangalore, India; 5 MVR Cancer Centre and Research Institute, Calicut, India

**Keywords:** PARP inhibitors, Maintenance recurrent ovarian cancer, Epithelial ovarian cancer, Olaparib, Niraparib, Rucaparib

## Abstract

Epithelial ovarian cancer (EOC) is usually diagnosed late at an advanced stage. Though EOC initially responds to treatment, the recurrence rate is pretty high. The efficacy of different targeted therapies reduces with each recurrence. Hence there is need of effective maintenance therapy in recurrent EOC. Recently, polyADP-ribose polymerase (PARP) inhibitors (PARPi) have been approved both for initial treatment of EOC and as its maintenance treatment. PARPi have also been found to act regardless of *BRCA* status or homologous recombination (HR) deficiency. Several trials testing PARPi early in maintenance therapy are in progress and their results will shed light on the optimal timing of maintenance therapy that gives the most benefit with least toxicity. Right patient selection for maintenance treatment is also a challenge. Hence, though PARPi are emerging as a promising maintenance treatment in recurrent EOC with prolongation of progression free survival (PFS), results from further trials and overall survival (OS) data from current trials are awaited to fulfill the gaps in understanding the role of this pathway in treatment of EOC. This review discusses the current therapies for EOC, challenges in the treatment of recurrent EOC, recent developments and trials in recurrent EOC maintenance with special focus on PARPi and future perspectives.

## Introduction

Globally, ovarian cancer (OC) is the seventh most common malignancy diagnosed among women and eighth leading cause of cancer mortality [1]. In 2018, United States is burdened with approximately 22,240 new cases and 14,070 deaths related to OC [2]. Among Indian women, ovarian cancer is the third leading cause of cancer after breast and cervical cancer. The age-adjusted incidence rates of OC vary between 5.4 and 8.0 per 100,000 persons in different parts of the country [3].

About 85–90% of all ovarian cancers are epithelial in origin, and around 70% of all epithelial ovarian cancers (EOCs) are high-grade serous (HGS) adenocarcinoma [4]. The term EOC often includes fallopian tube carcinoma and primary peritoneal carcinoma, as they have the same treatment and prognosis even though they are distinct from each other [4, 5].

Treatment after first-line therapy in EOC is contingent to platinum-free interval (PFI) and the side-effects from the previous therapy. Platinum-based chemotherapy (PBC) remains the mainstay of treatment in platinum sensitive disease (progression after 6 months of previous therapy). Many treatment options are being tried in recurrent EOC setting including targeted therapy with vascular endothelial growth factor (VEGF) inhibitors such as bevacizumab and tyrosine kinase inhibitors (TKI) such as pazopanib and nintedanib. While bevacizumab has shown benefit in recurrent setting, data on the efficacy of pazopanib and nintedanib in recurrent setting in platinum sensitive or platinum resistant disease (depending on whether progression occurred more than 6 months or less than 6 months after completing initial PBC is very limited) [6, 7].

Others, such as topotecan, pegylated liposomal doxorubicin (PLD), docetaxel, and etoposide have been investigated in platinum resistant disease.

However, if there is no clinical benefit after two lines of therapy, there is minimal likelihood of benefit from additional lines of therapy [6].

EOC is a challenge to treat because of its notoriety at recurrence with reduced benefit (< 20%), increasing toxicities, and poor quality of life with each subsequent line of therapy. Even with the newer therapies for recurrence, there has been no increase in progression free survival (PFS) after first-line therapy although overall survival (OS) is now longer [8]. Hence, there is an unmet need to optimize treatment in recurrent EOC. With increasing consensus that goals of therapy in recurrent EOC should be palliation of cancer-related symptoms, maintenance of quality of life, and extension of life, there is a lot of interest and research focusing on maintenance treatments for recurrent EOC [9]. In this concept, EOC is treated with maintenance dose therapy while the response to previous line PBC is still lingering. The concept is based on the hypothesis that advanced stage EOC usually has different clonal subpopulation of malignant cells of which some eventually become resistant to first-line chemotherapy, while others may respond to continuous maintenance dose therapy [10].

Poly ADP-ribose polymerase (PARP) inhibitors (PARPi) (olaparib, rucaparib, and niraparib) have emerged as one of the best approved target therapy options for recurrent EOC, especially in case of platinum-sensitive recurrent ovarian cancer (PSROC) [11–16]. This review will therefore focus on maintenance treatment of EOC with special emphasis on platinum sensitive disease and PARPi.

## Challenges in treating EOC

There is tremendous amount of research and focus on EOC as it presents in advanced stages and is the most fatal of the gynecologic malignancies. Since EOC does not have symptoms specific to cancer, there are no early screening and detection modalities [17, 18]. Thus, around 75% of women are diagnosed in advanced stage disease (FIGO IIIc or IV) [17, 18].

### Utility of laboratory markers in the diagnosis of EOC recurrence

Also, there are no effective lab tests known to monitor response to therapy in EOC. Though serum tumor marker CA125 is being used for initial diagnosis and for monitoring of response to chemotherapy, there is no evidence that a raised CA125 concentration alone can predict a survival benefit. Some patients with EOC present with rising CA125 levels despite having no signs and symptoms of recurrence. Treatment is a major challenge in these patients [19, 20]. Hence, there is need for other tumor biomarkers to assess the response to therapy.

### Recurrent EOC and common genetic mutations

Hereditary mutations in the *BRCA1* or *BRCA2* (*BRCA1/2*) genes occur more frequently in patients with platinum-sensitive EOC than platinum-resistant EOC [12], and these patients respond better to therapy and have greater survival benefits than women without the mutation [21]. However, routine *BRCA* testing and *BRCA* based treatment decisions are still at a nascent stage in India [22].

Newly diagnosed EOC is conventionally treated with de-bulking surgery and PBC in either neoadjuvant or adjuvant setting. However, even after being in full remission on first-line therapy, about 70–85% of patients with EOC relapse and median survival for patients with recurrent disease ranges from 12 months to 24 months [23]. Even with good response to treatment and survival after first recurrence on PBC, this treatment is rarely curative [23].

## Importance of platinum free interval in platinum sensitive relapsed ovarian Cancer

Upon recurrence in EOC, the choice of second-line chemotherapy is guided by the duration of response (DoR) to the prior PBC, also known as platinum-free interval (PFI), which is the time between completing initial PBC and progression. In patients with recurrent EOC, PFI is the most important predictor of response to subsequent lines of chemotherapy and the most important prognostic factor for PFS and OS. The longer the PFI, the higher the response rate (RR) and longer the duration of response [23].

### PFI and treatment responses in platinum sensitive relapsed EOC

Though patients with a PFI more than 6 months have been considered as platinum-sensitive, those with a PFI more than 12 months are considered definite or highly platinum sensitive, and those falling in the group with PFI of 6 to 12 months are now considered partially platinum-sensitive (PPS). However, treatment with platinum containing doublets in the PPS group gives unsatisfactory results with RR of only 25–30% to the subsequent PBC. The most effective regimen to be used in PPS is still uncertain and requires further research [7, 23–25].

It has been seen that with each recurrence the sensitivity and response to PBC decreases dramatically. Second-line PBC has a response of approximately 50–65% [25]. In a study, 51.6% of the patients showed clinical response to second-line therapy but the response dramatically reduced to only 11.9% in third-line chemotherapy [26]. Response profiles of late lines of non-platinum-containing regimens are in the range 10–15% with a PFS benefit of about 3 to 4 months, and OS benefit of around 12 months [27]. In three large European studies comprising of 1620 patients with OC, median PFS after the first, second, third, fourth, and fifth relapse was 10.2 [95% confidence interval (CI) 9.6–10.7], 6.4 (5.9–7.0), 5.6 (4.8–6.2), 4.4 (3.7–4.9), and 4.1 (3.0–5.1) months, respectively. Median OS after the first, second, third, fourth, and fifth relapse was 17.6 (95% CI 16.4–18.6), 11.3 (10.4–12.9), 8.9 (7.8–9.9), 6.2 (5.1–7.7), and 5.0 (3.8–10.4) months, respectively [28].

### Current status of selecting patients based on platinum sensitivity

Until recent years, recurrent EOC had no treatment option other than repeated courses of chemotherapy in second-line setting and beyond, with most patients eventually becoming resistant to PBC. Thus, selecting patients based on platinum sensitivity lost meaning after second-line therapy [25, 29, 30]. Hence, there is an unmet need for newer therapies in this area like PARPi and the concept of maintenance is gaining significance.

## Treatment options for platinum sensitive versus platinum resistant recurrent EOC

Guided by PFI, either chemotherapy or targeted therapies are used for EOC recurrence.

### Chemotherapy

Platinum sensitive patients are carefully selected for various combinations of PBC comprising of carboplatin or cisplatin in combination with paclitaxel, gemcitabine, PLD, or (with or without) bevacizumab [6, 7]. Combination therapy has been demonstrated to have better PFS and OS advantages over single platinum-agents [7, 23, 25].

If progression after first-line therapy occurs in less than 6 months after cessation of chemotherapy, the disease is considered platinum-resistant. During treatment EOCs can become platinum refractory, which means progression occurs during chemotherapy or within 1 month of cessation of chemotherapy [25, 29, 30]. The prognosis is poor for platinum-refractory and platinum-resistant patients. A non-platinum regimen is generally considered the most appropriate approach in these patients [23, 24]. In platinum-resistant patients, single agent non-platinum-containing therapies like PLD, paclitaxel, gemcitabine, or topotecan are recommended. Bevacizumab could be added in carefully selected patients [6].

In patients with partially sensitive recurrent EOC, the MITO-8 trial supports the use of PBC as platinum sensitive drugs prolong PFI and improve quality of life compared to non-platinum-based therapy [30, 31].However, when platinum is not an option due to anaphylaxis to platinum compounds, trabectedin (MITO15 phase II trial [NCT01772979]) or PLD with trabectedin (phase 3 OVA-301 study) can be considered. In these patients with anaphylaxis to platinum compound, platinum sensitive patients derived more benefit than platinum resistant patients and patients with *BRCA* mutation had longer PFS and OS [7, 32, 33].

### Targeted therapies

#### Approved therapies

Molecularly targeted inhibitors such as VEGF inhibitors (i.e., bevacizumab) and PARPi (i.e., olaparib, rucaparib, and niraparib) have emerged as treatment options in patients with advanced EOC after multiple prior lines of chemotherapy. Careful patient selection is required for bevacizumab and PARPi therapy, however, the criteria for patient selection are still evolving [25].

Bevacizumab, in combination with paclitaxel, PLD, or topotecan is approved for the treatment of patients with platinum-resistant recurrent EOC after maximum 2 prior lines of chemotherapy [27, 34]. Bevacizumab benefit has been demonstrated in platinum resistant setting in the AURELIA study [27]. In the platinum sensitive setting, bevacizumab has been tested in the OCEANS study and GOG-213 study [35–37]. Bevacizumab along with PBC like carboplatin-gemcitabine or carboplatin-paclitaxel is tested in MITO16 and MANGO-2 trials and in the phase III BOOST trial (NCT01462890) [31, 38, 39]. Bevacizumab monotherapy has also shown some clinical activity in platinum resistant patients with a clinical response rate of 16–21% in 1 to 3 prior lines of chemotherapy, and 13–16% in later lines of therapy [25].

PARPi have been the most studied, most effective, and least toxic in platinum sensitive recurrent EOC both as treatment and as maintenance [11]. These have been discussed in detail in a separate section.

#### Therapies under clinical development

TKIs like pazopanib (a multikinase inhibitor of VEGFR1–3, c-Kit and platelet-derived growth factor receptor α and β), trebananib (a fusion protein that selectively binds angiopoietin 1 and 2, preventing signaling through Tie-2 receptor), cediranib (a potent oral inhibitor of all 3VEGF receptor tyrosine kinases), nintedanib (a multi-kinase inhibitor); and immune checkpoint inhibitors like atezolizumab are being tested in various settings [7]. Success of targeted therapies lies on their specific inhibition of a driving molecule or pathway resulting in a tumor response rate of around 50–70% and a disease control rate of at least 80% [40].

Pazopanib, had demonstrated the PFS advantage in platinum-resistant and refractory OC (phase II trial: MITO-11; NCT01644825) [7, 30].

The immune checkpoint inhibitor atezolizumab in combination with PBC and bevacizumab is being tested in platinum sensitive setting in phase III randomized, double-blinded ATALANTE (NCT02891824) trial, and in AGO-OVAR 2.29 trial which is in a platinum resistant setting. Other combination trials include phase II study (EORTC-1508/NCT02659384) investigating atezolizumab with bevacizumab or acetylsalicylic acid and phase II/III study (NCT02839707) evaluating PLD with atezolizumab and/or bevacizumab [7].

## Maintenance therapy in recurrent EOC

A comprehensive analysis of 13 randomized placebo-controlled trials studying targeted maintenance therapy in OC showed that both PFS and OS improved compared to placebo (PFS: HR = 0.84, 95% CI 0.75 to 0.95, *p* = 0.001; OS: HR = 0.91, 95% CI 0.84 to 0.98, *p* = 0.02). However, treatment with targeted maintenance therapy was associated with increased incidence of adverse events (AEs) such as nausea, vomiting, diarrhea, fatigue, and hypertension which required dose reductions [41].

Though several maintenance therapies for recurrent OC have been tested, but only PARPi (niraparib, rucaparib, olaparib) have been approved by the FDA and/or European Medical Association (EMA) [7]. Paclitaxel has been tested in GOG 212 trial which failed to show OS benefit and demonstrated increased toxicities after complete response to first-line therapy [42]. Bevacizumab has been investigated in GOG 218, and NCT02022917 study with inconclusive evidence of benefit in maintenance and in ICON7 with no evidence of benefit in PFS or OS in first line maintenance except in a subset of high risk patients [43, 44]. Very few patients in high risk sub-set in ICON7 received bevacizumab in recurrent setting, and hence its use in maintenance in recurrent EOC is not known [45]. The review by Cortez et al. has shown that *PARP* inhibitors such as niraparib in NOVA trial, olaparib in SOLO-2; and rucaparib in ARIEL-3 trials have demonstrated positive results in their respective clinical trials [7].

Pazopanib monotherapy was evaluated in AGO-OVAR16 (NCT00866697) trial and though it showed improved median PFS versus placebo 17.9 vs 12.3 months, respectively in platinum-sensitive maintenance (hazard ratio [HR], 0.77; 95% CI 0.64 to 0.91; *p* = 0.0021), further studies are required to validate these findings as OS benefit has yet not been demonstrated [30, 46].

Nintedanib has been successfully studied in a phase 3 trial in combination with chemotherapy followed by maintenance monotherapy (AGO-OVAR 12/NCT01015118). Nintedanib demonstrated significant improvement in median PFS in the treatment group compared with control group (17.2 versus 16.6 months; HR = 0.84; 95% CI 0.72–0.98; *p* = 0.024) [47]. In their review, Elit & Hirte reported results of a phase 2 trial of nintedanib (NCT00710762) as maintenance in patients with resistant or partial PSROC which showed a higher PFS rate of 16.3% at 36 weeks compared with 5% in placebo group (HR = 0.68; 95% CI 0.44–1.07; *p* = 0.07) [48]. Evaluation of nintedanib in bevacizumab resistant, persistent, or recurrent EOC which does not progress for at least 6 months is in progress (NCT01669798) [7].

Preliminary data of pembrolizumab in combination with first-line PBC, followed by pembrolizumab maintenance showed promise in a small clinical trial of advanced OC presented at the 2018 Society of Gynecologic Oncology Annual Meeting [49]. Immune checkpoint inhibitors like atezolizumab are being tested in combination with bevacizumab and PBC in the ATALANTE (NCT02891824) trial [7].

Thus, both chemotherapy and non-PARPi targeted therapies (Table 1) either failed to give a conclusive benefit in EOC maintenance or the trials are still in progress with results awaited.

**Table 1:** Overview of investigational non-PARP inhibitors in EOC maintenance

Trial name [ClinicalTrials. gov identifier]	Drug	Mono/Combo; Phase	Patient population and key eligibility	N	Treatment arms	Primary endpoint	Results
GOG 212 [42]	Paclitaxel	Mono; Phase III	Advanced EOC in complete response	1157	Pac IV or CT-2103 (PP)	OS	No improvement in OS; increased toxicities
ICON 7 [44]	Bevacizumab	Combo; Phase III	Newly diagnosed early or advance EOC, first line	1528	C + Pac vs C + Pac + bev	PFS and OS	No improvement in PFS. No improvement in OS: restricted mean survival time chemo vs bev group 44.6 months (95% CI 43.2–45.9) vs 45.5 months (CI 44.2–46.7); log-rank (*p* = 0.85) Benefit only in high-risk subset: mean OS 34.5 months (95% CI 32.0–37.0) vs 39.3 months (37.0–41.7) (log-rank *p* = 0.03).
GOG 218 [43]	Bevacizumab	Combo; Phase III	Newly diagnosed advance EOC	1873	C + Pac (TC group) vs C + Pac + bev (TCP group) vs C + Pac + bev followed by bev maintenance	PFS and OS	PFS improvement of 3.8 months (10.3 for standard chemotherapy, 14.1 months for the maintenance regimen), Median OS was not significantly different between arms
NCT02022917 [43]	Bevacizumab	Combo; Phase II	Extensive stage IIIC or IV EOC	27	Postoperative PBC + adjuvant and maintenance bev	AEs	Ongoing; study completion Dec 2018
ATALANTE (NCT02891824)	Atezolizumab	Combo; Phase III	Late relapse EOC	405	Atezolizumab in combination with PBC + bev administered concurrent to chemotherapy and in maintenance	PFS	Ongoing; study completion 2023
AGO-OVAR16 (NCT00866697) [30, 46]	Pazopanib	Mono; Phase III	Platinum-sensitive maintenance in EOC	940	Pazopanib 800 mg OD maintenance in EOC patients who did not progress after one line of chemotherapy	PFS	Improved median PFS vs placebo 17.9 vs 12.3 months, respectively (HR: 0.77; 95% CI, 0.64–0.91; p = 0.0021)

*PP* paclitaxel poliglumex, *C* carboplatin, *Pac* paclitaxel, *PLD* pegylated liposomal doxorubicin, *PBC* platinum-based chemotherapy, *N* patient accural, *PFS* progression free survival, *OS* overall survival, *HR* hazard ratio, *CI* confidence interval, *TC* Chemotherapy, *TCP* paclitaxel + carboplatin + bevacizumab, *Bev* bevacizumab, *AE* adverse events, *EOC* epithelial ovarian cancer, *OD* Once a day

## Application of PARP inhibitors in the treatment of recurrent ovarian Cancer

**Fig. 1:**
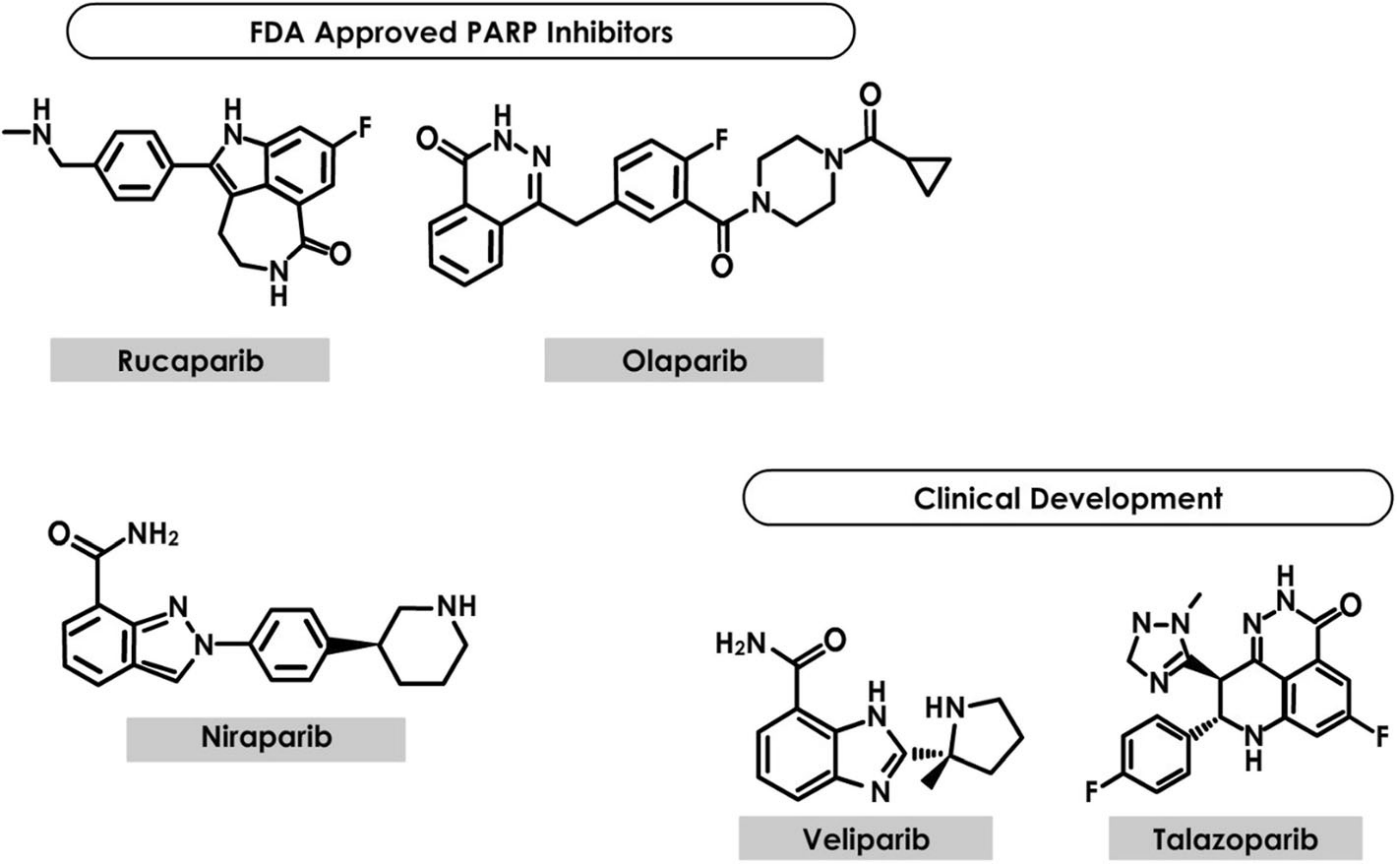
Structure of PARP Inhibitors

PARP (Fig. 1) includes a class of 17 enzymes that interrupt DNA repair, disrupt stability, and cause cell death by their action on single-strand DNA breaks or base excision repair (BER) [7].

**Fig. 2:**
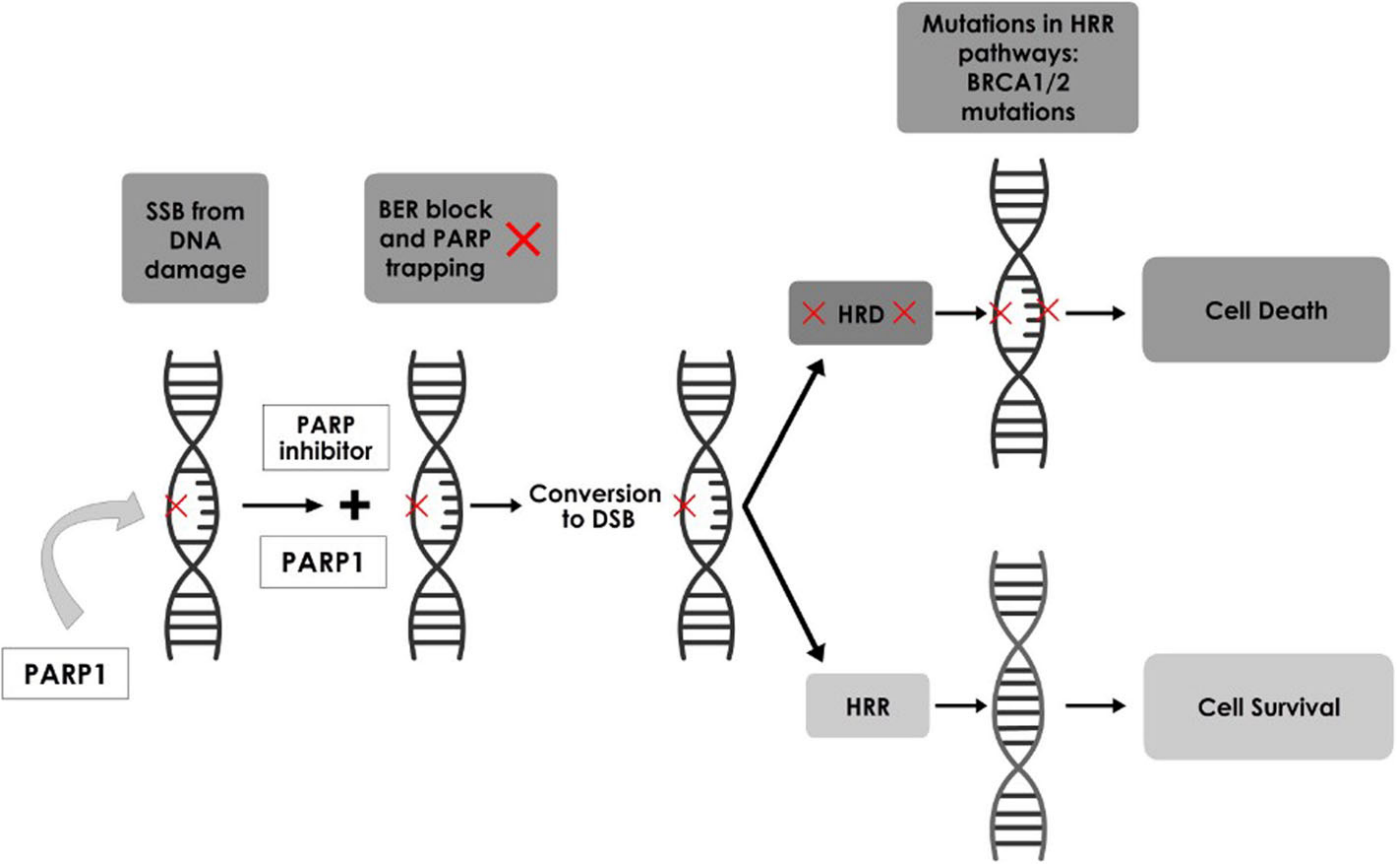
Mechanism of Action of PARP Inhibitors. Note: in the presence of a PARP inhibitor, *PARP*1 is activated by DNA damage (single strand break). BER is blocked and, on replication, a DSB is formed from the single strand break. In presence of functional If HRR (in normal healthy cells), DNA damage is repaired, and the cell survives. In cells with HRR deficiency (as seen in BRCA mutations), the break is either not repaired or repaired by error-prone NHEJ or MMEJ. This causes genomic instability and ultimately cell death. Footnote: BER: Base excision repair; BRCA: breast and ovarian cancer susceptibility gene; DSB: DNA double-strand breaks; HRD: HRR deficiency; HRR: homologous recombination repair; MMEJ: microhomology-mediated end-joining; NHEJ: nonhomologous end-joining; PARP: poly (ADP-ribose) polymerase

PARPi could be useful in HGS ovarian cancers (HGSOC) as they have detectable germline and somatic mutations. Another notable characteristic seen in many HGSOC is DNA methylation of genes (e.g. *BRCA*1/2) which participate in homologous recombination (HR) DNA repair during epigenetic silencing. Approximately 30% of HGSOCs have *BRCA*1/2 mutation and silencing which frequently cause diminished HR activity. HR DNA repair is a critical step in accurate repair of DNA (Fig. 2) following double strand break (DSB) [16, 40]. In addition, PARPi are selectively lethal in HR deficient (including *BRCA*1/2-mutated) cancers as they inhibit alternate DNA repair pathways such as BER and single-strand break repair [16, 40].

However, in patients with PSROC, olaparib has been shown to significantly improve PFS regardless of *BRCA* mutation and niraparib showed improved PFS regardless of *BRCA* mutation and homologous recombination deficiency (HRD) status [7, 16]. This indicates that apart from HR genes, there could be other potential predicative biomarkers and mechanisms for PARPi sensitivity. It also suggests that although efficacy of both olaparib and niraparib is highest in *BRCA*-mutated population, other patients may benefit too as improved PFS advantage was seen in wild-type *BRCA* positive patients with low genetic loss of heterozygosity (LOH) [7, 16].

The only accepted predictors of a response to PARPi are cisplatin sensitivity and presence of *BRCA*1 or *BRCA*2 mutations [7].. However, the optimal timing and duration of administration of PARPi is still a matter of debate. Based on data obtained from olaparib trials, it appears that the efficacy of PARP inhibition decreases with increasing lines of chemotherapy. This suggests that use of PARPi earlier in OC treatment may be more beneficial [50].

Though PARPi have side-effects including anemia, increase in creatinine, myelodysplastic syndrome (MDS) and acute leukemia (Table 2), they are usually not as severe as those observed with chemotherapy or with other targeted therapies and are easily managed with dose reductions and modifications. About, ≤1% patients in both olaparib and rucaparib studies have also developed MDS/acute myeloid leukemia, highlighting the need to monitor patients for hematologic toxicities [13, 14].

**Table 2:** Side effects of PARP inhibitors

		Olaparib SOLO2/ENGOT-Ov21 (*n* = 195) [51]	Niraparib NOVA/ENGOT-OV16 (*n* = 367) [52]	Rucaparib ARIEL2 (*n* = 204)/ARIEL3 (*n* = 374) [53, 54]	Veliparib (*n* = 50) [55]
Grade 3 and 4 Adverse Events	Hematological	Anemia 38 (18%) Neutropenia 8 (4%) Thrombocytopenia 2 (1%)	Anemia 93 (25.3%) Neutropenia 72 (19.6%) Thrombocytopenia 124 (33.8%)	Anemia 45 (22%)/70 (19%) Neutropenia 16 (7%)/25 (7%) Thrombocytopenia 5 (2%)/19 (5%)	Leukopenia 1 (2%) Neutropenia 1 (2%) Thrombocytopenia 1 (2%)
	Non-hematological	Fatigue 8 (4%) Abdominal pain 5 (3%) Nausea 5 (3%) Vomiting 5 (3%)	Hypertension 30 (8.2%) Fatigue 30 (8.2%) Abdominal pain 4 (1.1%) Nausea 11 (3.0%)	Elevated AST/ALT 25 (13%)/39 (10%) Fatigue 18 (9%)/25 (7%) Abdominal pain 5 (2%)/9 (2%) Nausea 9 (4%)/14 (4%)	Nausea 2 (4%) Metabolism/nutrition 1 (2%) Other investigations 6 (12%)
Serious Adverse Events	Total	35 (18%)	Total 110 (30%) [16]	ARIEL2: total 50 (25%) ARIEL3: total 78 (21%)	Total 12 (24%)
	Individual	Anemia 7 (4%) Abdominal pain 3 (2%) Intestinal obstruction 3 (2%)		ARIEL2: Intestinal obstruction 10 (5%) Anemia 9 (4%) ARIEL3: Anemia 16 (4%) Pyrexia 6 (2%) Vomiting 6 (2%) Intestinal obstruction 3 (1%)	
Dose Changes due to Adverse Events		Dose reductions 49 (25%) Discontinuations 21 (11%)	Dose reductions: 244 (66.5%) Discontinuations: 54 (14.7%)	ARIEL2: Dose reductions: 80 (39%) Discontinuations: 19 (9%) ARIEL3: Dose reductions: 203 (55%) Discontinuations: 50 (13%)	Dose Reductions: 31 (62%) Discontinuations: 31 (62%)^a^

In the treatment of advanced recurrent OC, PARPi continues to be tested in three distinct settings, especially after 2–3 or more prior lines of PBC [7, 11, 16]:

1. Monotherapy (olaparib in relapsed germline *BRCA* + ve EOC in SOLO3/ NCT02282020; rucaparib versus chemotherapy in *BRCA* + ve EOC in ARIEL4/NCT02855944)
2. Maintenance therapy (discussed in a separate section): (ENGOT-OV16 NOVA/NCT01847274, SOLO 2/NCT01874353, ARIEL3/NCT01968213)
3. Combination therapy (niraparib versus niraparib-bevacizumab ENGOTOV24/AVANOVA/NCT02354131; olaparib or cediranib maleate and olaparib in NCI-OVM1403/NCT02446600; olaparib with paclitaxel and carboplatin versus carboplatin and paclitaxel alone in patients with PSROC in NCT01081951

### PARP inhibitor Monotherapy in recurrent EOC (Table 3)

#### Approved therapies

In late 2014, olaparib received accelerated FDA approval as monotherapy for patients with advanced OC previously treated with ≥3 lines of chemotherapy and harboring deleterious or suspected deleterious germline *BRCA* (g*BRCA*) mutations. Olaparib was granted accelerated approval based on an objective response rate (ORR) of 34% (46/137; 95% CI 26–42) and median duration of response (DOR) of 7.9 (95% CI 5.6–9.6) months in a heavily pretreated (≥3 lines of therapy) 137 patients with advanced OC who had a germline *BRCA* mutation in Study 42 [56]. This accelerated approval was contingent upon verification of clinical benefit from two trials: SOLO 2 which studies olaparib in PSROC with a *BRCA*1/2 mutation who had received at least two lines of previous chemotherapy [51] and the phase 2 LIGHT study (NCT02983799) which assesses the efficacy and safety of olaparib in patient cohorts stratified by use of different HRD genetic tests [57].

**Table 3:** Overview of PARP inhibitor trials in EOC Treatment

Trial name [ClinicalTrials.gov identifier]s	PARPi (approval status)	Mono/Combo; Phase; PARP enzyme targeted	Patient population and key eligibility	N	Treatment arms	Primary endpoint	Results/Trial status
Study 42 (NCT01078662) [56]	Olaparib (approved)	Mono; *PARP* 1 > *PARP*2> > *PARP*3	Confirmed genetic *BRCA*1 and/or *BRCA*2 mutation	298	Olaparib 400 mg (8 × 50 mg capsules), oral BID until progression of the disease	Tumor response rate	ORR was 34% (46/137; 95% CI: 26–42) and median DoR was 7.9 (95% CI 5.6–9.6) months
LIGHT study (NCT02983799) [57]	Olaparib (approved)	*PARP* 1 > *PARP*2> > *PARP*3	*gBRCA*m and wt PSROC HGS	260	Olaparib 300 mg after ≥1 L PBC	ORR	Ongoing; study completion 2020
SOLO3/ NCT02282020 [11]	Olaparib (approved)	Mono; Phase III; *PARP* 1 > *PARP*2> > *PARP*3	g*BRCA*m PSROC	411	Olaparib vs. physician’s choice of single agent standard of care non-platinum based chemotherapy (TPC) after ≥2 L PBC	ORR	ORR 72% olaparib vs 51% TPC OR 2.53 (1.40, 4.58) *p* = 0.002 PFS 13.4 vs 9.2 mo HR 0.62 (0.43, 0.91); *P* = 0.013
NCT01081951	Olaparib (approved)	Combo; Phase II; *PARP* 1 > *PARP*2> > *PARP*3	PSROC (both germline *BRCA* and sporadic)	162	200 or 400 mg BID olaparib + C + Pac vs C + Pac as first line	PFS	Prelim results: Median PFS 12.2 (olaparib arm) versus 9.6 mos (no olaparib) *p* = 0.0012; study completion Dec 2018
NRG-GY005 (NCT0250226)	Olaparib (approved)	Combo; Phase II/III; *PARP* 1 > *PARP*2> > *PARP*3	Platinum resistant recurrent high-grade OC	680	Olaparib/cediranib versus single agent chemotherapy	PFS (phase II) OS (phase III)	Ongoing; study completion 2023
NCI-OVM1403/NRG-GY004 (NCT0244660)	Olaparib (approved)	Combo; Phase III; *PARP* 1 > *PARP*2> > *PARP*3	PSROC HGS; *BRCA* stratified	549	Olaparib versus olaparib/cediranib versus platinum doublet	PFS	Ongoing; study completion Dec 2019
ENGOTOV24/AVANOVA (NCT02354131)	Niraparib (investigational; approved only for maintenance)	Combo; Phase I/II; *PARP*1 and *PARP*2	PSROC	108	Niraparib versus niraparib-bevacizumab	PFS	Ongoing; study completion 2020
ARIEL 2 (Part 1: results available; *Part 2 ongoing*) (NCT01891344) [53]	Rucaparib (approved)	*PARP* 1>>>> > *PARP*2	PSROC HGS	493	600 mg BID rucaparib	PFS	Prelim results: PFS in the BRCAm (HR 0.27, 95% CI 0.16–0.44, *p* < 0.0001) LOH high (0.62, 0.42–0.90, p = 0.011)
ARIEL4/NCT02855944	Rucaparib (approved)	Mono; Phase III; *PARP* 1>>>> > *PARP*2	*BRCA*m HGSOC (platinum sensitive and resistant)	345	Rucaparib vs PBC	PFS	Ongoing; study completion 2024
NCT01113957	Veliparib (investigational)	Combo; *PARP* 1 and *PARP*2	Recurrent HGSOC (both germline *BRCA* and sporadic allowed)	168	[1] Veliparib and temozolomide vs. [2] PLD	ORR	Results not available
NCT01306032 [58]	Veliparib (investigational)	Combo; *PARP* 1 and *PARP*2	Recurrent HGSOC (both germline *BRCA* and sporadic allowed)	75	Oral cyclophos 50 mg + veliparib 60 mg daily vs. Oral cyclophos 50 mg daily	ORR	Well tolerated with some clinical activity; addition of veliparib at 60 mg daily did not improve either the response rate or the median PFS

*PARPi*: Poly ADP-ribose polymerase inhibitor; PLD: pegylated liposomal doxorubicin; Cyclophos: cyclophosphamide; CI: confidence interval; DoR: duration of response; N: patient accural; wt: wild-type BRCA; gBRCAm: BRCA germline mutation carrier; BRCAm: BRCA mutant; PFS: progression free survival; ORR: overall response rate; HGSOC: high-grade serous ovarian cancer; PSROC: platinum sensitive recurrent ovarian cancer; HR: hazard ratio

SOLO 3 is an ongoing phase III randomized trial of olaparib vs. physicians’ choice of four chemotherapy options in patients with platinum-sensitive germline BRCA mutant HGSOC, who had two or more lines of PBC [7, 11]. The results of the SOLO3 were recently presented at ASCO 2019 by Penson and colleagues showed a statistically significant and clinically meaningful ORR and PFS benefit with olaparib versus non – platinum based chemotherapy.

In late 2016, rucaparib, another PARP inhibitor was approved as monotherapy for patients with PSROC that progressed with ≥2 lines of chemotherapy and harboring deleterious *BRCA* mutation (germline or somatic). The accelerated approval was based upon positive results from ARIEL2 Part 1 study, which demonstrated significantly longer PFS in the *BRCA* mutant (hazard ratio [HR] 0.27, 95% CI 0.16–0.44, *p* < 0·0001) and LOH high (0.62, 0.42–0.90, *p* = 0.011) subgroups compared with the LOH low subgroup. Part 2 of the ARIEL2 trial is ongoing (NCT01891344) [14, 53, 54]. Ongoing studies (Study 10 Part 2B, ARIEL2 Part2, ARIEL4) are evaluating rucaparib treatment in both platinum sensitive and resistant patient population [59].

### PARP inhibitors combination therapy in recurrent EOC (Table 3)

Various olaparib or veliparib combinations with chemotherapy have been tried as it was thought that BER disruption via *PARP* inhibition would synergize with chemotherapy [58, 60]. However, when PARPi are combined with chemotherapy, full doses of both regimens are rarely achieved as they both have overlapping myelosuppressive toxicities [61].

Hence PARPi combinations with biologics like antiangiogenics or PI3 kinase pathway inhibitors or immune checkpoint inhibitors and other biologic agents are under way, especially for HSGOC, in the hope of inducing greater DNA damage and HRD with lesser toxicities than chemotherapy combinations [11]. Phase I studies of olaparib in combination with the PI3K inhibitor BKM120 (NCT01623349) and the AKT inhibitor AZD5363 (NCT02208375) have demonstrated evidence of activity in OC [62, 63].

Olaparib alone or in combination with cediranib was tested in a phase 2 study in platinum-sensitive recurrent HGSOC (both germline and sporadic BRCA positive) (NCT01116648). Median PFS was 17.7 months for cediranib and olaparib group (*n* = 44) versus 9.0 months for olaparib group (*n* = 46; HR = 0.42; *p* = 0.005). Though OS data were not mature at the time of reporting, the combination group demonstrated a trend toward longer OS [7, 11, 64]. Based on these positive results, two phase III trials are underway in both platinum sensitive and platinum resistant OC. Olaparib or cediranib alone or in combination compared with standard chemotherapy is being tested in platinum-resistant EOC in a randomized phase II/III trial, NRG-GY005 (NCT02502266). Olaparib versus olaparib/cediranib versus platinum doublet in *BRCA* stratified platinum sensitive recurrent HGSOC is being tested in NRG-GY004 (NCT02446600) trial. The phase III PAOLA – 1 trial (see Table 4) (NCT02477644) is evaluating a combination of Olaparib and Bevacizumab maintenance post platinum-based chemotherapy in 1st line Ovarian cancer irrespective of BRCA status [11, 66].

**Table 4:** Overview of ongoing trials of PARP inhibitors in maintenance

Trial name [ClinicalTrials. gov identifier]	Drug (approval status)	Mono/Combo; Phase; PARP enzymes targeted	Patient population; newly diagnosed/recurrent and key eligibility	N	Treatment arms	Primary endpoint	Results/Trial status
SOLO-2 (NCT01874353) [51]	Olaparib (approved)	Mono; Phase III; *PARP* 1 > *PARP*2> > *PARP*3	PSROC HSG; ≥2 PBC *BRCA*m only, g*BRCAmut* ***BRCA*1**: Ola:132 (67%) PBO: 61 (62%) BRCA2: Ola: 58 (30%) PBO: 35 (35%)	295	Olaparib 300 mg bid vs placebo maintenance after ≥2 PBC	PFS	Ola vs PBO: 19.3 months (95% CI 16.5–27.3) vs 5.5 months (5.0–5.8); HR [in favor of ola] 0.33, 95% CI 0.24–0.44; *p* < 0.0001).; study completion 2021
SOLO1 (NCT01844986) [11, 65]	Olaparib (approved)	Mono; Phase III; *PARP* 1 > *PARP*2> > *PARP*3	Germline *BRCA* mutant only, newly diagnosed HGSOC or endometrioid stage III and IV Of 391 patients at interim analysis, centrally confirmed g*BRCA*1/2 m: 388 somatic *BRCA*1/2 m: 2	451	Olaparib 300 mg bid vs placebo maintenance after 1 L PBC	PFS	Primary analysis: 60% vs. 27% in ola vs PBO (HR for disease progression or death, 0.30; 95% CI, 0.23 to 0.41; *p* < 0.001) Ongoing; study completion 2023
PAOLA1/ENGOT/GCIG (NCT02477644) [66]	Olaparib (approved)	Combo; Phase III; *PARP* 1 > *PARP*2> > *PARP*3	Newly diagnosed HGSOC	612	Bev or bev/olaparib maintenance after 1 L platinum/taxane/bev	PFS	Ongoing; study completion 2022
ICON 9 trial (NCT03278717)	Olaparib (approved)	Combo; Phase III; *PARP* 1 > *PARP*2> > *PARP*3	PSROC	618	Olaparib or olaparib/cediranib maintenance after 1 L PBC	PFS and OS	Ongoing; study completion 2023
ENGOT-OV26/PRIMA (NCT02655016) [67]	Niraparib (approved)	Mono; Phase III; *PARP*1 and *PARP*2	HRD positive, stage III and IV	305	Niraparib vs placebo maintenance after 1 L PBC with PR or CR	PFS	Ongoing; study completion August 2019
GOG-3005 (NCT02470585) [11]	Velaparib (investigational)	Combo; Phase III; *PARP* 1and *PARP*2	Advanced HGSOC, both *BRCA* germline mutation carriers and *BRCA* wild types	1140	Veliparib vs placebo maintenance after 1 L (C + Pac) or (C + Pac + veliparib)	PFS	Ongoing; study completion 2020

*PARPi* Poly ADP-ribose polymerase inhibitor, *C* carboplatin, *bev* Bevacizumab, *Pac* paclitaxel, *PLD* pegylated liposomal doxorubicin, *PBC* platinum-based chemotherapy, 1 *L* first-line, *N* patient accural, *wt* wild-type BRCA, *gBRCAm* BRCA germline mutation carrier, *BRCAm* BRCA mutant, *PFS* progression free survival, *ORR* overall response rate, *OS* overall survival, *HGSOC* high-grade serous ovarian cancer, *PSROC* platinum sensitive recurrent ovarian cancer, *HRD* homologous recombination deficiency, *HR* hazard ratio, *CI* confidence interval, *bid* twice daily, *od* once daily, *nira* niraparib, *ruca* rucaparib, *PBO* placebo, *PR* partial response, *CR* complete response

## Third-line and beyond in EOC – the unmet need

Patients with EOC having 3 or more recurrences have few effective treatment choices. Thus, several clinical trials are investigating treatment options in EOC for these patients. Various single and combination agents are being tried in late stage trials namely PARPi (ricaparib, niraparib, and olaparib), NUC-103 (gemcitabine prodrug), mirvetuximab soravtansine (an antibody-drug conjugate targeting the folate-alpha receptor), trabectedin (novel alkylating chemotherapy agent), birinapant (SMAC mimetic and IAP inhibitor), and volasertib (Plk1 inhibitor) [25].

Ovarian cancer with *BRCA*1/2 mutations can also recur after ≥3 lines of therapy. *BRCA*1/2 mutant EOC has shown susceptibility to *PARP* inhibitors due to synthetic lethality (Table 5). In a phase 2 study (Study 42, NCT01078662), olaparib has shown positive results in germline *BRCA*1/2 mutant positive OC patients with ≥3 recurrences and stratified by platinum sensitivity [56]. Olaparib demonstrated ORR of 46, 30 and 14% in patients considered platinum sensitive (but not considered suitable to receive further PBC), platinum resistant, and platinum refractory, respectively. ORRs with olaparib were in the range of 50–57% and 31–39% for platinum-sensitive and platinum-resistant patients, respectively. Patients who received ≥6 prior lines of therapy had much lower ORR of 20 and 13% for platinum-sensitive and platinum-resistant patients, respectively. Median PFS was 9.4 months (95% CI 6.7–11.4) and 5.5 months (95% CI 4.2–6.7) in platinum-sensitive and platinum-resistant patients, respectively [56].

**Table 5:** *PARP*i Maintenance by HRD Status

Study	Phase	Patient population	Treatment arms	HR classification	PFS/OS/ORR
Study 19 [12, 68]	II	Platinum-sensitive recurrent serous ovarian cancer; ≥2 PBC with PR or CR to most recent PBC	olaparib 400 mg bid vs PBO	*Germline or tumor BRCA* Ola: 74 (56%) vs PBO 62 (50%)	**PFS**: ola: 11.2 months (*BRCA*m)/7.4 months (wt) p < 0.00001, For PBO: 4.3 months (*BRCA*m)/5.5 months (wt); *p* = 0.007 **OS**: *BRCA*m ola vs PBO 34.9 months (95% CI 29.2–54.6) vs 30.2 months (23.1–40.7); HR 0.62 (95% CI 0.41–0.94) nominal *p* = 0.025*BRCAwt* in11 (15%) of 74 patients with *BRCA*m who received ola maintenance for ≥5 years: ola vs *PBO:* 24.5 months (19.8–35.0) vs 26.6 months (23.1–32.5); HR 0.83 (95% CI 0.55–1.24); nominal *p* = 0.37
SOLO1 (NCT01844986) [11, 65]	III	Newly diagnosed HGSOC or endometrioid stage III and IV	olaparib 300 mg bid vs PBO	Of 391 patients at interim analysis, centrally confirmed g*BRCA*1/2 m: 388 somatic *BRCA*1/2 m: 2	PFS: Primary analysis: 60% vs. 27% in ola vs PBO (HR for disease progression or death, 0.30; 95% CI, 0.23 to 0.41; p < 0.001)
SOLO-2 [51]	III	PSROC HSG; ≥2 PBC	olaparib 300 mg bid vs PBO	g*BRCAmut* ***BRCA*1:** Ola:132 (67%); PBO: 61 (62%) ***BRCA*2:** Ola: 58 (30%); PBO: 35 (35%)	In 286 patients with *BRCA*1/2 (Ola: 190; PBO: 96). median PFS Ola vs PBO (19.3 months [95% CI 16.5–27.3] vs 5.5 months [5.0–5.8]; HR [in favor of ola] 0.33, 95% CI 0.24–0.44; *p* < 0.0001).
ENGOT-OV16/NOVA [52]	III	PSROC	Niraparib 300 mg vs. PBO o.d	*BRCA*1/2 positive & *BRCA*1/2 negative g*BRCA* cohort: 203 (niraparib: 138,PBO: 65) non-g*BRCA* cohort: 350 (niraparib: 234, PBO: 116	Median PFS g*BRCA*m 21 mo (nira) vs 5.5 months (PBO) (HR, 0.27; 95% confidence interval [CI], 0.17 to 0.41), *p* < 0.00001; Non-g*BRCA*m HRD+ 12.9 months (nira) versus 3.8 months (PBO) (HR 0.38 95% CI, 0.24 to 0.59), *p* < 0.00001; All Non-g*BRCA*m 9.3 months (nira) versus 3.9 months (PBO) (HR .45; 95% CI, 0.34 to 0.61), p < 0.00001
ARIEL3 (NCT01968213) [54, 69] Ongoing; completion June 2020	III	PSROC- HSGOC, ≥2 prior PBC with PR or CR to most recent PBC	Rucaparib maintenance therapy 600 mg p.o. b.i.d. vs. PBO	HRD stratification at the time of enrollment *(BRCAmut; BRCAwt/* LOH high; *BRCAwt/* LOH low) BRCAm ruca: 130 [35%] vs PBO: 66 [35%] HRD carcinoma ruca: 236 [63%] vs PBO: 118 [62%])	Median PFS in *BRCA-mut* rucaparib vs PBO 16.6 mo (95% CI 13.4–22.9) vs. 5.4 mo (95% CI 3.4–6.7) (HR 0.23 [95% CI 0.16–0.34]; p < 0.0001) HRD carcinoma ruca vs PBO: 13.6 mo (10.9–16.2) vs 5.4 mo (5.1–5.6; 0.32 [0.24–0.42]; *p* < 0.0001)

Abbreviations: *AE* adverse event, *bid.* twice daily, *od* once daily, *p.o.* orally or per mouth, *chemo* chemotherapy, *HRD* homologous recombination deficiency, *HR* hazard ratio, *IV* intravenous, *LOH* loss of heterozygosity, *mut* mutated, *N/A* not available, *mo* months, *ORR* overall response rate, *PARP* Poly (ADP-ribose) polymerase, *PFS* progression-free survival, *po.* by mouth, *wt* wild type, g*BRCA* germline BRCA mutation, non-g*BRCA* non-germline *BRCA* mutation, *PSROC* platinum sensitive recurrent ovarian cancer, *HR* hazard ratio, *PBC* platinum based chemotherapy, *Ola* olaparib;nira: niraparib; ruca: rucaparib, *PBO* placebo, *PR* partial response, *CR* complete response

The results of these trials may open new treatment opportunities for EOC with unmet needs. Also, the concept of maintenance therapy is likely to prolong time to recurrence by treating with therapies approved in this setting during the window when the response to previous line chemotherapy is still lingering.

To provide benefit in this setting, US FDA has accepted the concept of maintenance therapy and has been granting accelerated approvals to drugs for both recurrent EOC and for maintenance therapy setting [25].

## Role of PARP inhibitors in maintenance therapy

### Approved therapies (Table 5)

All 3 PARP inhibitors - olaparib, niraparib and rucaparib have been approved as maintenance monotherapies in patients with PSROC [13, 15, 51].

#### Olaparib

Of all the PARPi, olaparib has been the most extensively tested molecule in the clinical setting with favorable results [51]. Olaparib is a potent PARPi that is selectively cytotoxic to cells, blocks PARP from repairing damaged single-strand DNA breaks and preserves repair of efficient cells expressing deleterious *BRCA1/2* [70*]*.

The accelerated olaparib approvals came based on positive results from phase 3 SOLO2 and phase 2 Study 19 [12, 51]. SOLO2 investigated olaparib maintenance after ≥2 lines of chemotherapy for OC patients with germline *BRCA* mutations. The study demonstrated that olaparib significantly improved PFS (Investigator assessed) compared to placebo (19.1 months vs. 5.5 months; HR = 0.30 [95% CI 0.22–0.41], *p* < 0.0001) with marked improvement in quality of life. The most common AEs of grade 3 or worse severity were anemia (19% in olaparib group vs 2% in the placebo group), neutropenia (5% vs 4%) and fatigue or asthenia (4% vs 2%). Serious adverse events were experienced by 18 and 8% of patients in the olaparib and placebo group, respectively [51]. OS results have not yet been reported [51, 71]. In the phase 2 Study 19 (AZ19/NCT00753545), in patients with OC irrespective of BRCA status, olaparib increased PFS compared with placebo (median PFS 8.4 months versus 4.8 months; HR = 0.35; 95% CI 0.25–0.49; *p* < 0.0001) [12].

The phase III SOLO-1 trial evaluated the efficacy of olaparib as maintenance therapy in patients with newly diagnosed advanced HGSOC with *BRCA*1, *BRCA*2, or both (*BRCA*1/2) mutations who had a complete or partial clinical response after PBC. The recently published results of SOLO-1) demonstrated that the olaparib group had 70% lower risk of disease progression or death than with placebo group after a median follow-up of 41 months. The Kaplan–Meier estimate of the rate of freedom from disease progression and from death at 3 years was 60% in olaparib and 27% in the placebo group (PFS NR vs 13.8 months in placebo arm; HR for disease progression or death, 0.30; 95% CI, 0.23 to 0.41; *p* < 0.001) [65]. The olaparib arm had an unprecedented estimated 36 month benefit over placebo arm in PFS. The median time to the first subsequent therapy or death was 51.8 months vs 15.1 months in the olaparib vs placebo group, respectively (HR, 0.30; 95% CI, 0.22 to 0.40) and this benefit seemed to be maintained in the PFS2 as well, in spite of the fact that 35% of the patients in the placebo arm received a post progression PARP inhibitor (31% maturity, HR, 0.50; 95% CI, 0.35 to 0.72; p < 0.001) [65].

Since 2016, the NCCN [3] has included olaparib in its guidelines as a fourth-line treatment for women with EOC who carry a deleterious *BRCA1/2* gene (both germline and somatic). In February 2018, the approval for use as maintenance treatment came regardless of patients’ *BRCA* mutation status thus increasing its reach to a wider set of population [6, 72].

In 344 patients with newly diagnosed germline BRCA mutant HGSOC or endometrioid stage III and IV, olaparib versus placebo maintenance is being tested early post PBC in SOLO1 (NCT01844986) trial [11].

#### Niraparib

The accelerated niraparib approvals came based on positive results from the phase 3 NOVA study which compared response in germline *BRCA (gBRCA)* mutation positive and negative patients. While patients on both groups showed significant benefit, those in the g*BRCA* cohort, showed significantly longer PFS than placebo [52]. OS results have not yet been reported [50]. Approximately two thirds of patients did not have germline *BRCA* mutations. PFS (by BICR) in the germline *BRCA* mutations versus placebo was 21.0 months vs 5.5 months (*p* < 0.0001). In the group with non-mutated *BRCA* but with HRD positive score versus placebo, PFS was 12.9 months vs 3.8 months (p < 0.0001). PFS was longer in niraparib-treated patients (6.0 versus 3.9 months, *p* = 0.02) even in the group without mutations and HRD negative. In patients with germline *BRCA* mutations versus those without mutations, niraparib reduced the risk of progression or death by 74% (HR = 0.26) vs 55% (HR = 0.45). Majority of AEs were hematological and could be successfully managed by dose modification. Thus, niraparib use is independent of *BRCA* status and HRD score [50, 52].

Additionally, early use of niraparib versus placebo as maintenance post-response to first-line PBC is being tested in 305 patients with HRD positive, stage III and IV newly diagnosed OC in the ENGOT-OV26/PRIMA (NCT02655016) trial [67].

#### Rucaparib

On April 6, 2018 FDA approved rucaparib as maintenance therapy for recurrent EOC [14]. Approval was based on the ARIEL3 study with similar enrollment criteria as olaparib and niraparib maintenance therapy studies. However, the enrollment was not dependent on *BRCA* status. In the phase III ARIEL3 [NCT01968213] trial, the median PFS of maintenance rucaparib versus placebo group was 10.8 months vs 5.4 months. The median PFS was higher in patients with germline or somatic *BRCA* mutations: 16.6 months (95% CI 13.4–22.9) vs 5.4 months (95% CI 3.4–6.7) in maintenance rucaparib versus placebo group, respectively. The PFS was 13.6 months (HR = 0.32, *p* < 0.0001) in the HRD-group (including *BRCA* mutant or *BRCA* wild type/LOH-high) and 10.8 months (HR = 0.37, p < 0.0001) in the “intent to treat” group (including *BRCA* mutant, *BRCA*-wild type and LOH-low, indeterminate or high) [73] These patients also experienced a 77% reduction in the risk of progression or death with rucaparib versus placebo (HR, 0.23; 95% CI 0.16–0.34; *p* < .0001). To determine the HRD status, the FDA concurrently approved the complementary diagnostic test, FoundationFocus™ CDx *BRCA* LOH [54, 59].

Foote et al. (2018) assessed the relative value of maintenance therapies and biomarkers in PSROC and presented the results at the 49th Society of Gynecologic Oncology (SGO) Annual Meeting on Women’s Cancers in March 2018. The value of each drug and biomarker in maintenance setting of EOC was validated using the American Society of Clinical Oncology (ASCO)‘s Net Health Benefit (NHB) and the European Society of Medical Oncology (ESMO)‘s Magnitude of Clinical Benefit Scale (MCBS). The following drugs and trials were examined: Study 19, NOVA, SOLO2, and ARIEL3 trials for PARPi, OCEANS, and GOG 213 trials for bevacizumab treatments, ICON6 for cediranib [74].

In germline/somatic-*BRCA* mutation cohorts being treated with maintenance PARPi had the highest ASCO scores: olaparib (SOLO2) = 47, (Study 19) = 62; niraparib (NOVA) = 50; rucaparib (ARIEL3) = 54. ESMO scores were also high for maintenance PARPi in germline/somatic-BRCA mutation cohorts. Low value scores were seen for bevacizumab, cediranib, and wild-type *BRCA* [74].

Based on their efficacy and safety profile and ease of oral administration PARPi are emerging as a potential maintenance therapy in recurrent EOC with response to ≤2 prior lines of PBC [11].

However, despite their advantage as a safe and efficacious maintenance treatment in EOC, PARPi are costlier than the other available therapies. PARPi are 18.8, 6.9, and 2.2–2.7 times costlier than paclitaxel, pembrolizumab, and bevacizumab, respectively. Hence, it is important to carefully select the patients and optimize the timing of PARPi maintenance in order to give the most adequate response [75].

### PARP inhibitor therapies in clinical development (Table 3)

Veliparib, an oral *PARP*1 and *PARP*2 inhibitor has demonstrated a response rate of 26%, with a median PFS of 18.8 months in phase 2 study as monotherapy in relapsed germline *BRCA* mutant OC, but results need to be validated in larger phase 3 trials [55].

Talazoparib, another oral *PARP*1 and *PARP*2 inhibitor was studied in phase I trials and showed some clinical activity in relapsed g*BRCA* mutant OC. However, currently it is not being actively pursued [11].

### PARP inhibitors for maintenance therapies in clinical development (Table 4)

In women with PSROC, maintenance olaparib in combination with cediranib is currently being tested in ICON 9 trial (NCT03278717). Various combination trials testing early use of maintenance therapy in newly diagnosed OC in response to first-line therapy are underway. The GOG-3005 (NCT02470585) trial is testing veliparib versus placebo maintenance therapy following carboplatin and paclitaxel or carboplatin, paclitaxel, and veliparib in 1100 patients with advanced HGSOC, both BRCA germline mutation carriers and BRCA wild types.

## Summary

There is adequate data to support maintenance treatment in recurrent EOC, and the use of PARPi in the treatment (olaparib and rucaparib) and maintenance (olaparib, rucaparib, and niraparib) setting for platinum-sensitive HGSOC regardless of *BRCA* status or HR deficiency in relapsed platinum sensitive ovarian cancer. The recently reported results of SOLO – 1 marks the foray of olaparib as an effective maintenance therapy in newly diagnosed gBRCA positive ovarian cancer patients who are in CR or PR to 1st line surgery and platinum-based chemotherapy. The AEs with all the PARPi can be majorly managed with dose interruptions or modifications. Olaparib is the most studied safe and efficacious treatment and maintenance therapy in the platinum sensitive setting. Though many combination therapies with PARPi are under trial, there is no trial-based comparison among these three approved PARPi. Hence the relative efficacy or toxicity of the individual drugs is not known. Several trials testing PARPi early in maintenance therapy are in progress and their results will shed light on the optimal timing of maintenance therapy that gives the most benefit with least toxicity. Right patient selection for maintenance treatment is also a challenge. Hence, though PARPis are emerging as a promising maintenance treatment in recurrent EOC with prolongation of PFS, results from further trials are awaited to fulfill the gaps in understanding the role of this pathway in treatment of EOC.

## Data Availability

Not applicable.
